# P303: Safety of intravenous samples and risk management of infectious waste: the case of Mali

**DOI:** 10.1186/2047-2994-2-S1-P303

**Published:** 2013-06-20

**Authors:** AM Traoré, M Fomba, T Cissé, S Yehia, B Diarra, DS Ouloguem, H Cissé, DK Minta

**Affiliations:** 1 Service of Infectious Diseases, University Hospital of Point G, Bamako, Mali

## Introduction

We conducted a one-day survey in a teaching hospital, two district centers, a community health center and military dispensary in Bamako in February 2012, as part of a multicenter GERES study.

## Objectives

We aimed to determine the materials used in intravenous (IV) blood collection to clarify the availability of safety equipment and describe the measure to eliminate infectious waste.

## Methods

Safety equipment for collection of IV blood samples were available in any center. The blood was drawn by syringes + needles and the equipment for collect blood was chosen by different people on the center. Funding was provided by the health department or the patient.

## Results

A policy for waste management existed in every site but only 3 sites out of 5 had a written procedure, generally not distributed in all services. Containers for waste recovery were not specific (collector card, plastic security bin bag and a recycled bottle of bleach). Cardboard boxes were available for free. Two centers conducted soaking in bleach before handling. Four centers of five or had a dedicated staff trained in waste disposal. The cremation was carried out in three sites out of 5. Other centers sent their waste to the health service district of Bamako.

## Conclusion

Unavailability of safety equipment contributes to the exposure of personnel to blood exposure. We must strengthen the technical platform and the system improves sorting and waste management.

## Disclosure of interest

None declared

